# Nanoantennas Patterned by Colloidal Lithography for Enhanced Nanophosphor Light Emission

**DOI:** 10.1021/acsanm.2c03258

**Published:** 2022-11-11

**Authors:** Jose M. Viaña, Manuel Romero, Gabriel Lozano, Hernán Míguez

**Affiliations:** Instituto de Ciencia de Materiales de Sevilla, Consejo Superior de Investigaciones Científicas-Universidad de Sevilla, C. Américo Vespucio 49, Sevilla41092, Spain

**Keywords:** plasmonics, patterning, nanosphere lithography, rare-earth nanoparticles, transparent thin films, photoluminescence, localized surface plasmon resonance (LSPR), local density of optical states (LDOS)

## Abstract

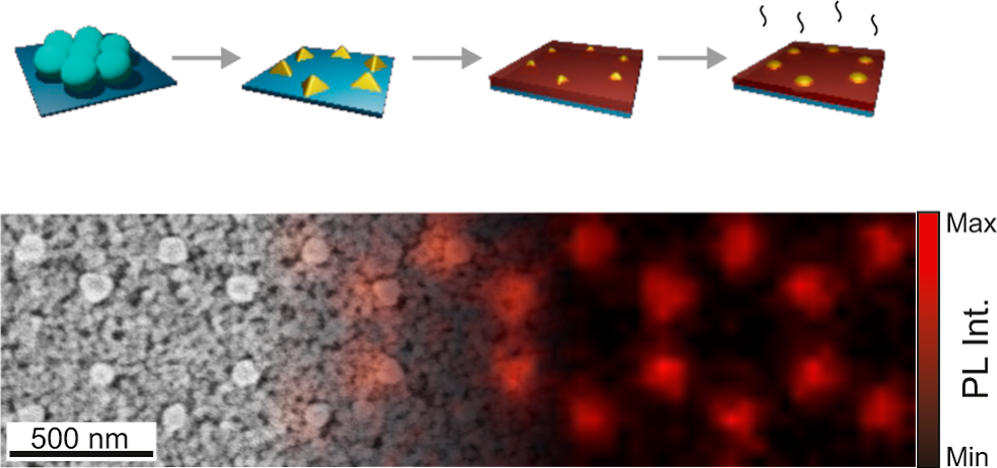

Transparent coatings made of rare-earth doped nanocrystals, also known as nanophosphors, feature efficient photoluminescence and excellent thermal and optical stability. Herein, we demonstrate that the optical antennas prepared by colloidal lithography render thin nanophosphor films with a brighter emission. In particular, we fabricate gold nanostructures in the proximity of GdVO_4_:Eu^3+^ nanophosphors by metal evaporation using a mask made of a monolayer of polymer beads arranged in a triangular lattice. Optical modes supported by the antennas can be controlled by tuning the diameter of the polymer spheres in the colloidal mask, which determines the shape of the gold nanostructure, as confirmed by numerical simulations. Confocal microscopy reveals that metallic antennas induce brighter photoluminescence at specific spatial regions of the nanophosphor film at targeted frequencies as a result of the coupling between gold nanostructures and nanophosphors. Patterning of nanophosphor thin layers with arrays of metallic antennas offers an inexpensive nanophotonic solution to develop bright emitting coatings of interest for color conversion, labeling, or anti-counterfeiting.

## Introduction

Phosphors are key materials for light emission because they feature high conversion efficiencies along with thermal and chemical stability.^1−3^ For this reason, these photoluminescent materials have been extensively studied and are widely employed in solid-state lasers, fluorescent lamps, light-emitting diodes, displays, or solar cells.^4−9^ However, increasingly demanding specifications are pushing the development of novel phosphors with tailored properties of interest for smart illumination devices from general lighting to horticulture or healthcare.^10−14^ In this context, nanocrystals made of inorganic matrices doped with rare-earth (RE) elements, the so-called phosphor nanoparticles or nanophosphors, have gained relevance in recent years not only for their use as nanomarkers for biotechnology^15,16^ but also because they allow the fabrication of transparent thin coatings of interest for security, labeling, or optoelectronics.^17−21^ Phosphor nanosizing has also opened the door to the development of phosphor nanoparticle-based optical materials, in which photonic architectures and phosphor thin films are combined to tune RE emission properties with an unprecedented precision.^22−24^

Among the different photonic strategies employed to enhance the emission of nanomaterials,^25,26^ metallic nanostructures that support localized surface plasmon resonances (LSPRs) have demonstrated great potential.^27,28^ LSPRs originate from the collective oscillations of free electrons located on the surface of the metal when it is illuminated.^29^ Metallic nanoparticles feature large scattering cross sections and may provide resonant photoexcitation and/or enhanced radiative decay for emitters whose position spatially overlaps with the field profile of the LSPR at a frequency that spectrally matches the excitation or emission band of the emitter, respectively.^30^ In the particular case of phosphor coatings, many studies report on the combination of metal nanoparticles randomly dispersed with phosphor powders or phosphor films that yield few-fold emission enhancement due to the LSPR coupling.^31−37^ Nevertheless, the properties of the LSPR are mainly determined by the size and shape of the metal nanoparticle.^38^ For this reason, it is central to achieve a precise control of the fabrication of the plasmonic nanostructure and its relative position with respect to the emitting layer. A wide variety of lithographic techniques can be used to this end, including (i) scanning lithography, in which the nanometer-scale structures are directly written by focusing a beam of electrons or ions in a resist layer;^39^ (ii) optical lithography, in which the periodic patterns are created in a resist by multiple laser interference;^40^ or (iii) soft lithography, in which a film is grafted using a nanostructured stamp.^41^ In general, these techniques provide the nanostructures with a fine precision and enable the emission enhancement of emitters located nearby.^42−45^ However, nanofabrication techniques typically include some costly steps and their compatibility with the phosphor processing is challenging. Thus, there are limited successful demonstrations of such techniques directly applied to the phosphors. Indeed, in a very recent example, it was shown that a nanophosphor thin film monolithically patterned with a square array of nanoholes using soft lithography yields a twofold directional enhancement of the emitted light.^46^

Colloidal lithography, also known as nanosphere lithography, allows preparing large-area nanostructures in an inexpensive way using a monolayer of self-assembled spheres as an evaporation mask.^47^ In the particular case of phosphor materials, this method has been employed to pattern thick and thin yttrium aluminum garnet phosphor layers to enhance the emission.^48,49^ Known for decades, this method is widely used for the fabrication of periodic arrays of metallic nanostructures due to its simplicity and versatility, as well as its low cost compared to the other lithographic techniques.^50^ However, the combination of optical antennas attained by colloidal lithography and thin phosphor films has not been reported in the field.

In this work, we present a simple method to enhance the emission of a transparent phosphor (GdVO_4_:Eu^3+^) nanoparticle thin film (∼40 nm) using resonant gold nanostructures fabricated by colloidal lithography. As an evaporation mask, we employ a monolayer of polystyrene (PS) spheres arranged in a triangular lattice. Controlling the dimensions of the mask and the processing conditions allows tuning the LSPR of gold nanostructures to be resonant with the main radiative transition of Eu^3+^. High-temperature processing is not always compatible with nanophotonics, which brings to light a trade-off. For this reason, it is challenging to demonstrate plasmonic-mediated enhancement of RE efficient emission. In our work, we found the preparation conditions to demonstrate brighter coatings of thin phosphor nanoparticle films that are already efficient using gold antennas. In particular, we demonstrate that the PL of phosphor nanoparticles located in the vicinity of the nanoantennas is enhanced by ∼12-fold as our microspectroscopic analysis reveals. Numerical simulations confirm that the origin of the PL increase is the enhanced coupling of the emission of the Eu^3+^ mediated by the LSPR supported by each individual antenna. Our results show that the plasmonic decoration of transparent phosphor layers by colloidal lithography represents an inexpensive nanophotonic design that yields bright ultrathin light-emitting coatings, which may be of interest for color conversion, sensing, or security.

## Methods

### Nanophosphor-Coated Antenna Array Preparation

In order to fabricate the array of metal nanostructures, first we deposit an ordered PS sphere monolayer via a wedge evaporation method.^51^ It starts from an aqueous suspension of PS spheres with a concentration of 2.1% vol. The substrate is placed at a 3° angle with respect to the horizontal, and 300 μL of the suspension is left for 2 days between 20 and 30 °C and at 90% humidity as water evaporates. Two different sphere size diameters were used, namely, 560 and 720 nm. Then, a 100 nm-thick Au layer is deposited via thermal evaporation with a Univex 250 vacuum coating system. We chose Au for stability reasons since gold is far less reactive than silver, for instance, at high temperatures. To complete the process, PS spheres are removed by sonication in absolute ethanol for 2 min. Then, a film of ∼40 nm of GdVO_4_:Eu^3+^ phosphor nanoparticles is deposited over the array by spin coating under the following conditions: two depositions at 2000 rpm for 1 min. GdVO_4_:Eu^3+^ nanoparticles (∼40 nm in size) were previously prepared following a synthesis reported elsewhere.^52^ Finally, the film over the array was annealed at 450 °C for 30 min.

### Structural Characterization

Resulting photonic structures were inspected using a Hitachi S-4800 high-resolution scanning electron microscope.

### Optical Characterization

Reflectance and transmittance spectra were measured using a UV–vis–NIR spectrophotometer (Cary 5000, Agilent Technologies) coupled to a universal measurement accessory (UMA).

### Photoluminescence Characterization

Emission spectra and time-dependent PL intensity were measured with an Edinburgh FLS1000 spectrofluorometer under an excitation of λ_ex_ = 276 nm. Time-dependent PL measurements were registered for the most intense Eu^3+^ emission band at 620 nm. Absolute photoluminescence quantum yield (PLQY) measurements were performed in an integrating sphere using FLS1000. Our films were excited at 285 nm, and the emission and scattering peaks measured in the integrating sphere in the spectral range comprised between 270 and 850 nm. In addition, the scattering and emission peaks of a scattering sample were also measured to serve as a reference. Spatial-resolved microscopic PL measurements were obtained using a confocal optical microscope (Leica Stellaris 8) using an oil immersion objective and 465 nm laser light as excitation source. Samples were illuminated through a glass coverslip employed as the substrate. Spatial resolution is diffraction limited (ca. 250 nm × 250 nm). The step size of the scanning is 70 nm. Emitted photons were collected in the wavelength range comprised between 606 and 636 nm that corresponds to the main emission band of GdVO_4_:Eu^3+^. Background measurements were taken in the wavelength range comprised between 780 and 810 nm.

### Modeling

The refractive index of the nanophosphor film was obtained by fitting the reflectance and transmittance spectra of a 250 nm-thick reference sample. Results are shown in the Supporting Information. Reflectance, transmittance, and field intensity distributions were calculated using Lumerical, a commercial software, based on the finite difference in time domain (FDTD) method. Plane wave illumination at normal incidence, perfectly matched layer (PML) conditions at the upper and lower boundaries, and periodic boundary conditions at the lateral boundaries of the unit cell were used in all simulations. The fourfold symmetry of our systems was also considered. The substrate was assumed to be semi-infinite and dielectric (*n* = 1.51), while a complex refractive index was used for the PS spheres [Re(*n*) = 1.58; Im(*n*) = 0.01],^53^ and *n* = 1.3 was considered for the nanophosphor film. Ballistic transmittance, that is, zeroth-order transmittance, and specular reflectance at each incident wavelength were extracted from the zeroth diffraction order making use of the specific field monitors.

## Results and Discussion

**Figure 1:**
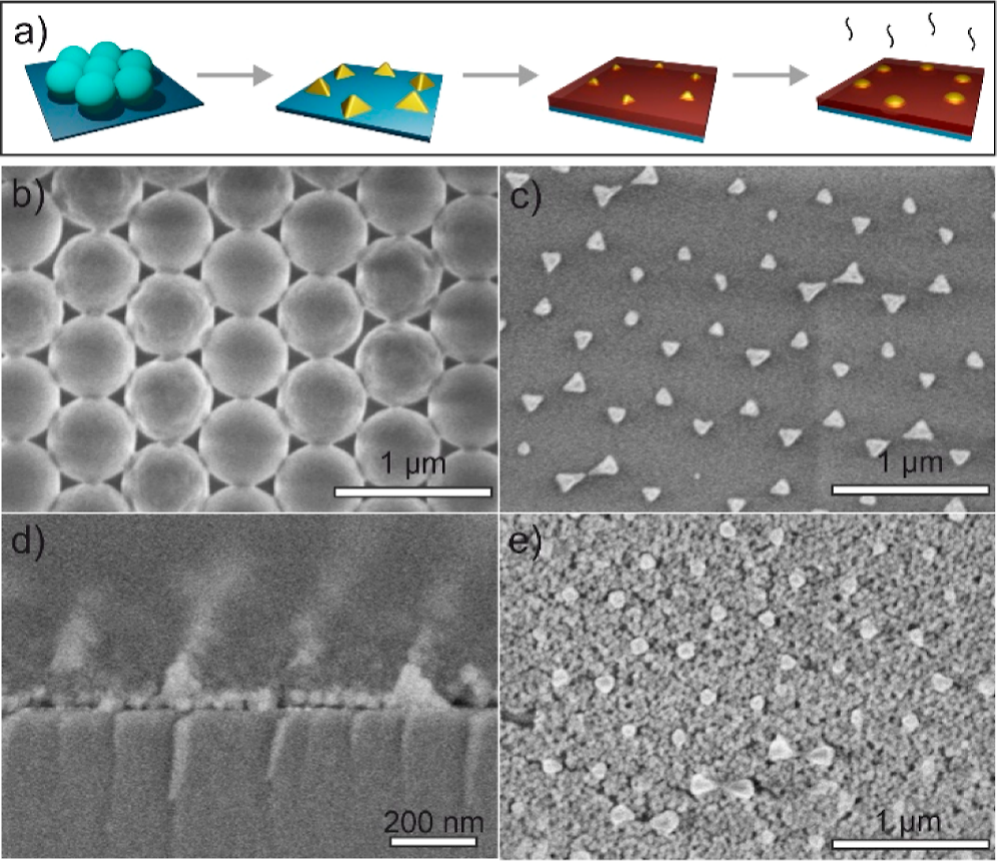
(a) Schematic description of the fabrication process of gold nanoislands coated with phosphor nanoparticles. It includes (i) preparation of a colloidal mask made of a monolayer of spheres, (ii) gold evaporation over the mask and mask removal, (iii) nanophosphor film deposition, and (iv) thermal treatment. Scanning electron micrographs of different steps of the process. (b) Top view of a monolayer of PS spheres with *D* = 560 nm. (c) Top view of the array of gold nanoislands. (d) Cross view of the gold nanostructures with phosphor nanoparticles. (e) Top view of the nanophosphor film over the gold nanoislands after annealing at 450 °C.

The fabrication of an array of gold nanostructures by colloidal lithography involves a series of steps as detailed in the Methods Section and illustrated in Figure 1a. In brief, we first prepare our colloidal mask that is made of an ordered monolayer of submicron PS spheres using a wedge evaporation method. Under specific experimental conditions, as the liquid evaporates, spheres accumulate in the vicinity of the three-phase contact line and form a high-quality colloidal monolayer on the substrate. Figure 1b shows a scanning electron microscopy (SEM) image of such a colloidal crystal film, in which it can be observed that the spheres self-organize in a triangular lattice with the periodicity of the sphere diameter (*D*). Notice that the number of nanoislands and their size and shape are given by the diameter of the PS spheres in the colloidal mask since the amount of gold that can be deposited is proportional to the volume between the PS spheres in the mask. As a matter of fact, as *D* enlarges, the dimensions of the islands increase, but their density over the substrate reduces. In particular, we fabricate gold nanoislands using colloidal masks with *D* = 560 nm and *D* = 720 nm (see Supporting Information). We deposit a thin layer of gold by thermal evaporation and remove the PS mask. As a result, a periodic array of gold nanostructures arranged in a honeycomb lattice is created, where nanoislands sit at the vertices of each hexagon in the network, as observed in Figure 1c. Next, we deposit a thin layer of GdVO_4_:Eu^3+^ nanophosphors by spin coating over the nanoislands, as it can be observed in Figure 1d. SEM shows that the phosphor nanoparticles coat the substrate uniformly, with a film thickness of ∼40 nm. It also reveals the tetrahedral shape of the nanoislands, with a height of ∼100 nm. Finally, we anneal the films at 450 °C in order to improve the brightness of Eu^3+^ cations. It is well known that as-prepared nanophosphors feature low PLQY due to the presence of organic ligands in the surface of the nanoparticles and limited crystallinity associated to the preparation conditions. Thermal processing removes surface quenchers and lattice defects and improves crystallinity,^19,22^ which results in a significant increase of the PLQY up to 55% (see Supporting Information). Nevertheless, thermal processing also modifies the shape of the nanoislands, as shown in the SEM picture displayed in Figure 1e. Indeed, gold nanostructures appear rounded, with the edges of the tetrahedra almost completely blurred—see the Supporting Information.

**Figure 2:**
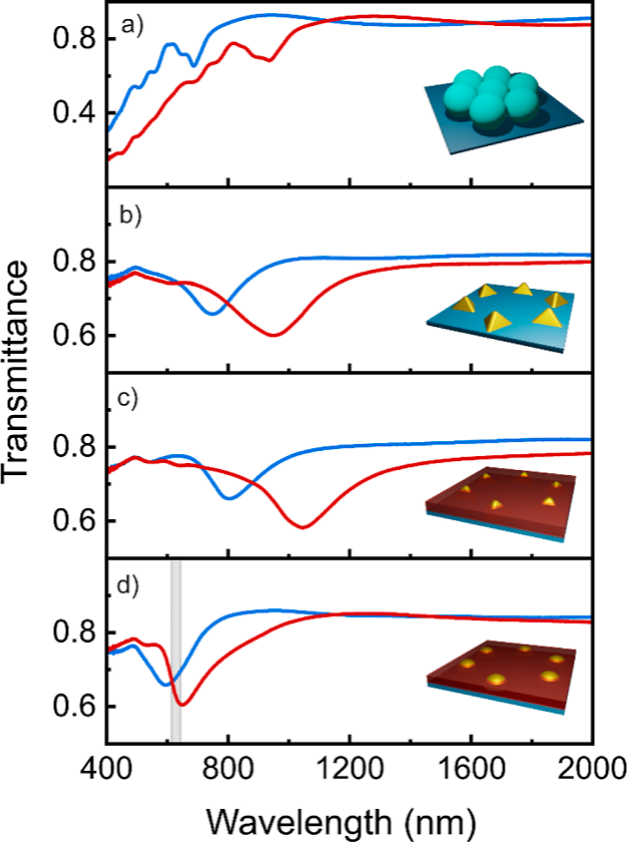
Experimental ballistic transmittance spectra of the (a) colloidal mask, (b) periodic array of gold nanostructures, (c) same array after GdVO_4_:Eu^3+^ nanophosphor thin film deposition, and (d) after thermal processing. Gray band in (d) depicts the main emission band of GdVO_4_:Eu^3+^. Blue (red) line corresponds to samples prepared using a colloidal mask made of polymer spheres with *D* = 560 nm (720 nm). 3D sketches of the different structures are shown as insets.

Figure 2 shows ballistic transmittance (*T*) spectra measured from the fabricated samples at each step of the processing. Reflectance measurements are included in the Supporting Information. First, we show the transmittance of the colloidal mask in Figure 2a as a function of the wavelength of the incident light (λ). Features shift spectrally to higher frequencies when *D* reduces as it is expected for an ordered monolayer of dielectric spheres. In particular, the main feature appears as an abrupt drop of intensity at  and a transmission band at , which is associated to the excitation of light modes in a colloidal crystal slab in the so-called high-energy range.^53^ Also, the low-frequency regime shows a high transparency window (*T* > 85%) modulated by low-intensity secondary lobes related to Fabry–Perot oscillations. After metal deposition and mask removal, *T* of the periodic array of gold tetrahedra is shown in Figure 2b. *T* reduces throughout the spectrum due to the presence of a metal. More interestingly, a broad dip is clearly observed at λ = 748 nm for the sample made out of smaller PS spheres (*D* = 560 nm) that shifts to λ = 949 nm for the one prepared out of 720 nm spheres, which we associate to the excitation of a plasmon resonance in the gold nanostructures. *T* also shows a much weaker feature at λ = 616 nm that shifts to λ = 530 nm for the smallest spheres, which originates from a LSPR, as simulations will confirm. Furthermore, the extinction is higher for the biggest nanoislands as large nanostructures typically show higher scattering cross sections. No major change in the optical response is observed when a phosphor nanoparticle thin film is deposited atop the metal array. As displayed in Figure 2c, we only observe a small shift of the resonances to lower frequencies due to a slight increase in the effective refractive index of the medium surrounding the top of the gold nanoparticles because of the nanophosphors. In turn, annealing drives a significant change of the optical response of the material—see Figure 2d—, which we associate to the modification of the shape of the nanoislands. Indeed, thermal processing rounds the edges of gold tetrahedra, resulting in half spheres that feature resonant modes at different frequencies. As a matter of fact, transmittance minima appear at λ = 596 nm for *D* = 560 nm and λ = 650 nm for *D* = 720 nm. As a result, we have prepared arrays of gold half spheres that support LSPRs, which overlap spectrally with the main emission band of GdVO_4_:Eu^3+^—see gray shaded area in Figure 2d.

**Figure 3:**
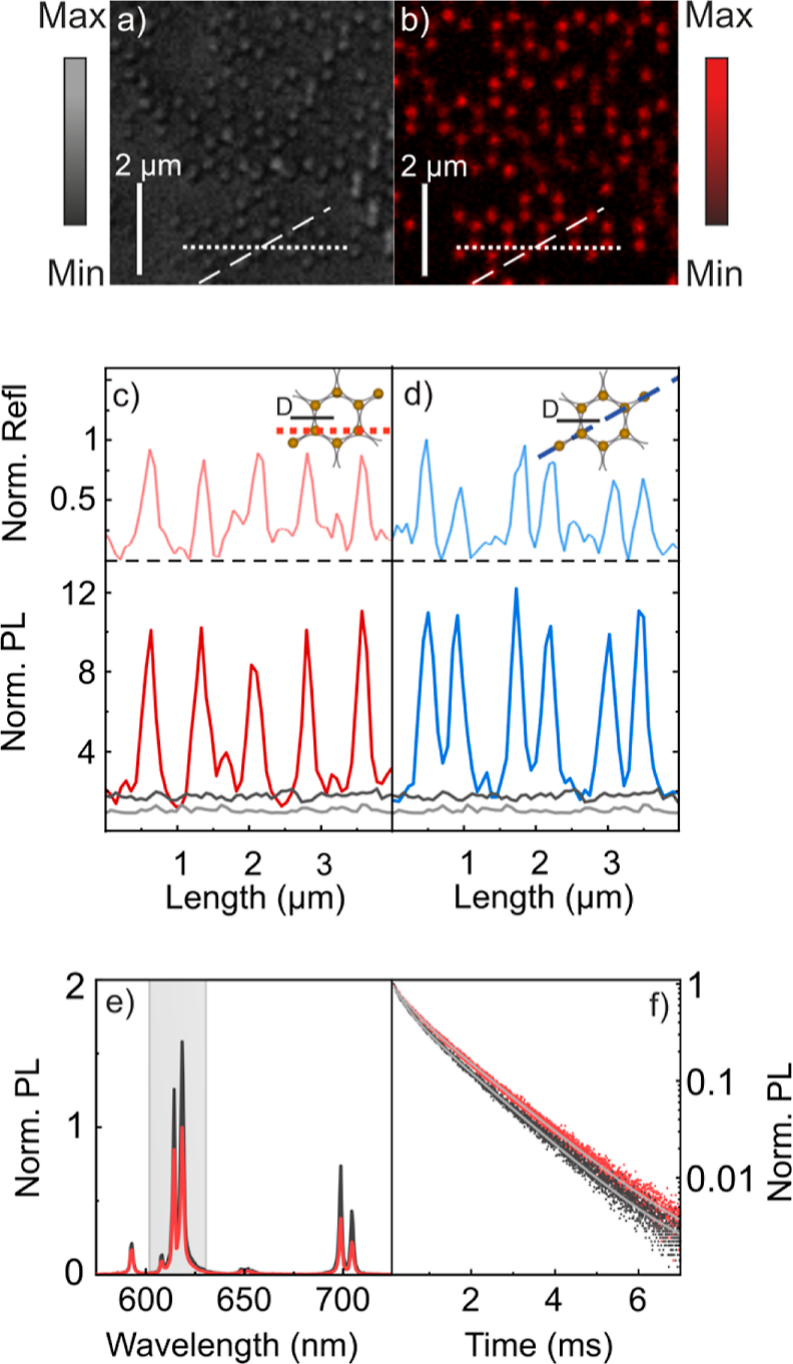
(a) Confocal scanning microscopy image of the reflection and (b) PL of a GdVO_4_:Eu^3+^ phosphor nanoparticle thin film deposited over an array of gold nanostructures. Light is integrated in the spectral range comprised between λ = 606 nm and λ = 636 nm. Dotted and dashed lines depicted the main directions of the lattice. (c,d) Spatial profile of the reflectivity (upper panel) and PL (lower panel) along the lines shown in (a,b). Black curves correspond to the PL of a nanophosphor thin film devoid of any gold nanostructure. Gray curves correspond to the emission profiles along the same directions in a spectral range where nanophosphors do not emit (between 780 and 810 nm). All profiles are normalized to the maximum intensity value of the profile measured from the reference sample. (e) PL intensity spectra measured from an area of 15 mm^2^ from a thin nanophosphor film deposited over the gold array (black curve) and a flat substrate, which act as a reference (red). Both spectra are normalized to the maximum intensity value of the reference sample. (f) Time-dependent PL monitored at λ = 620 nm from a thin nanophosphor film deposited over the gold array (black dots) and a flat substrate (red dots). Fittings are also shown as light gray curves.

In what follows, we analyze the photophysical properties of the nanophosphor film deposited atop the array of gold nanostructures. We have chosen the gold array fabricated using the colloidal mask with *D* = 720 nm to show the plasmon-mediated enhancement of the nanophosphor emission as a result of the coupling with LSPRs supported by the gold array. In particular, we take advantage of the high spatial resolution provided by the confocal scanning PL microscope. Indeed, reflectivity measurements displayed in Figure 3a, which are collected every 70 nm with a spatial resolution of 250 nm, reveal the spatial position of gold half spheres in the honeycomb lattice shown in Figure 1c, although some nanostructures were lost during the mask removal step of the processing. Figure 3b shows PL measurements recorded from the same region of the sample using a λ = 465 nm laser source for excitation. Results indicate that the phosphor nanoparticles whose position spatially overlaps with that of the gold nanostructures emit significantly brighter. Indeed, in Figure 3c,d, we plot spatial profiles of PL (thick colored curve) and reflectivity (thin color curve) along the two main directions of the array—see dotted and dashed lines in Figure 3a,b and insets of Figure 3c,d—. Most intense peaks for both reflectivity and PL are separated a distance ∼*D* in Figure 3c; also, the periodicity of each doublet in Figure 3d is ∼·*D*, as expected for this particular lattice geometry. Intensity profiles show PL or reflectivity integrated along the main emission band of GdVO_4_:Eu^3+^, that is, between λ = 606 nm and λ = 636 nm. We also show the intensity profile in a spectral region where nanophosphors do not emit (light gray curve) to estimate our background. For comparison, we display the PL intensity profile taken from a film of similar thickness devoid of any gold nanostructure (dark gray curve), which serves as the reference. Notice that all the intensity profiles are normalized to the maximum intensity value recorded for the reference sample. Thus, our results indicate that the phosphor nanoparticles placed in the vicinity of metal nanostructures feature a maximum 12-fold emission enhancement compared to a reference film without a metal. The physical origin of PL enhancement in a nanophotonic system is generally twofold. On the one hand, a photonic nanostructure can be resonant at the excitation frequency of the emitter, being the power absorbed by the emitter proportional to the field intensity enhancement at the pumping frequency. On the other hand, the spontaneous emission rate can be enhanced if the nanostructure supports an optical mode at the emission frequency for emitters whose position overlaps spatially with the field profile of the mode. LSPRs typically feature large field intensity enhancement values in close vicinity of the metal–dielectric interface. For this reason, the LSPR-mediated enhancement values depend strongly on the area from which the light is collected. Indeed, the maximum value of PL enhancement is obtained when light is collected from the smallest area considered, which corresponds to ∼0.05 μm^2^. This enhancement factor is gradually reduced when the scanning area increases, reaching 3.9-fold, and 2.75-fold when the PL is integrated over 0.9 or 25.0 μm^2^, respectively—see Supporting Information—. Notice that these values tend to that attained when the sampled area is ∼15 mm^2^ (1.57-fold enhancement), as shown in Figure 3e. In order to further support the plasmonic origin of the emission enhancement observed, we perform time-dependent PL measurements on an area of ∼15 mm^2^. Figure 3f displays intensity decay curves for a thin film deposited over an antenna array (red dots) and for a reference film of similar thickness devoid of metal nanostructures (gray dots). In both cases, we record the intensity at λ = 620 nm to monitor the ^5^D_0_ → ^7^F_2_ level of Eu^3+^ activators. A double exponential model is employed to fit the experimental data
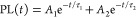
1where *A*_1_ and *A*_2_ are the amplitudes of each exponential component and τ_1_ and τ_2_ are the characteristic time of each component. The long decay component is typically associated to cations located in the bulk of the phosphor nanoparticles, whereas the short one is attributed to cations that sit in the surface of the nanocrystals. In another interpretation, these components account for the homogeneous and inhomogeneous effects, respectively. All fitting parameters are included in the Supporting Information. Our results indicate that the long component of the decay remains barely unchanged regardless of the metal nanostructures. However, we observe a significant reduction of the short PL(*t*) component (0.31 ms vs 0.36 ms) that we attribute to the presence of metallic nanostructures. Gold half spheres, thus, act as nanoantennas, which enable an LSPR-mediated enhancement of the emission of phosphor thin films.

**Figure 4:**
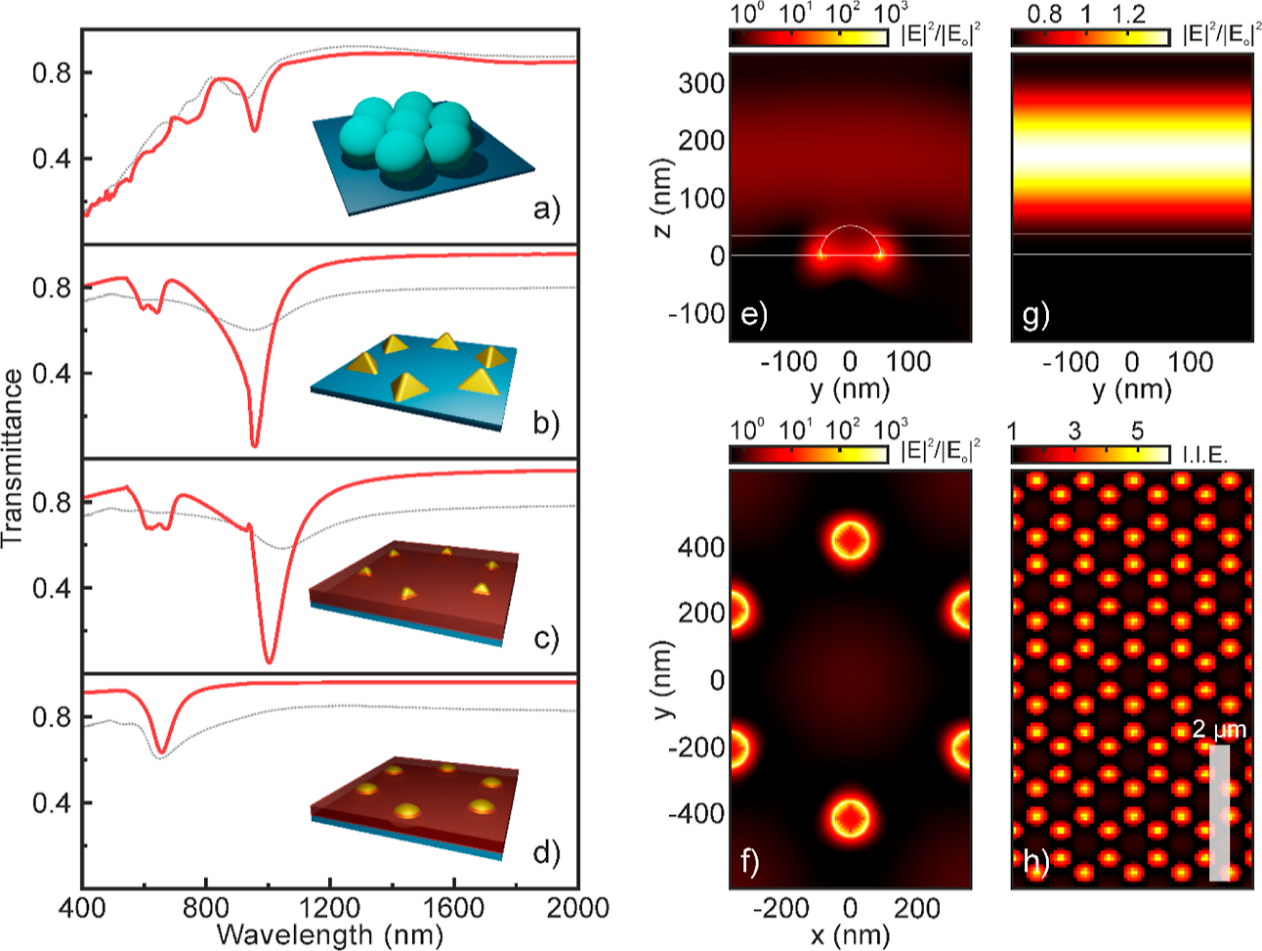
FDTD simulated ballistic transmittance spectra of a (a) monolayer of spheres of *D* = 720 nm and *n* = 1.58–*i*0.01, (b) periodic array of gold tetrahedra with a triangular base, 95 nm high and 225 nm on a side, (c) same array with a layer of 35 nm of dielectric with *n* = 1.3, and (d) periodic array of 104 nm diameter gold hemispheres covered by a 35 nm dielectric layer with *n* = 1.3. 3D sketches of simulated structures are shown as insets. (e, f) Simulated spatial distribution of the near-field intensity in an array of gold hemispheres on a substrate covered by a dielectric film in a plane intersecting the antennas at *x* = 0 (e) and *z* = 0 (f) in a unit cell of the array. (g) Simulated spatial distribution of the near-field intensity in the same system devoid of the antennas at *x* = 0. (h) Simulated spatial distribution of the IIE. Antennas and the different dielectric interfaces are outlined using gray curves.

In order to shed more light on the physical origin of the emission enhancement observed, we perform numerical simulations of light reflected and transmitted by the array of metal nanostructures and the spatial distribution of the total electric field intensity enhancement, that is, near-field intensity normalized by the incident intensity. We consider a plane wave incident in the direction normal to the array. Figure 4a–d shows the simulated transmittance for different steps of the processing. Fair agreement is found when comparing the experimental (black curves) and calculated (red curves) spectra. Figure 4a displays the calculated transmittance spectrum of a monolayer of PS spheres arranged in a triangular lattice. We introduce an imaginary part of the refractive index of the PS spheres—Im(*n*) = 0.01—to reproduce the experimental response of such a photonic crystal slab.^53^ Then, we analyze the influence of the size and shape of the gold nanostructure in the optical response of the array. In particular, we consider an array of triangular-based tetrahedra of 225 nm side and 95 nm height. We also assume that the volumes have rounded edges and vertices since this fact has an impact on the spectral position of the resonance. Calculations show two bands of low transmission, that is, one of large intensity centered at λ = 1002 nm, along with a shallower one at λ = 640 nm. Simulations of the spatial distribution of the electric field intensity at these wavelengths—see Supporting Information—reveal the localized character of the resonances, with the field primarily enhanced in the proximity of gold nanostructures, as expected for an LSPR. In particular, the latter can be associated to individual antennas, whereas the former relates to the interaction between nearest neighbors in the lattice. Indeed, if we calculate the response of an array of tetrahedra with the same dimensions but arranged in a lattice with larger periodicity such that the distance between tetrahedra increases, the spectral position of the resonances remains barely unchanged, but the mode at λ = 1002 nm reduces its strength drastically—see Supporting Information. We reproduce the spectral position of the main extinction band, finding fair agreement between the calculated and experimental transmittance spectra for the array of gold nanostructures, as shown in Figure 4b. However, we observe some deviations related to the spectral width of the resonance and its extinction intensity, which we attribute to the defects and a certain distribution of the sizes and shapes of the nanoantennas. Figure 4c displays the calculated transmittance spectrum of the gold array with a thin layer (*n* = 1.3) atop to account for the nanophosphors. Simulations reproduce the red shift of the spectrum (40 nm) that we observe experimentally when phosphor nanoparticles are deposited over the optical antennas. Finally, we simulate the response of the metal array when thermal processing modifies the shape of the gold nanostructures, rounding the edges of the tetrahedra into hemispheres. As a result, the main feature appears at λ = 656 nm, which originates from the excitation of in-plane dipolar resonances at each half sphere. Notice that the mode that the tetrahedra arrays show at longer wavelength is not present in this system since hemisphere dimensions prevent nearest-neighbor coupling for this periodicity. As expected from an LSPR, field intensity is mainly enhanced in the metal–dielectric interface in the vicinity of the gold nanostructure with no field enhancement extending in the space between the antennas, as shown in Figure 4e,f. See the Supporting Information for a direct comparison between the response of tetrahedra and half spheres. Optical and structural properties of resonant nanostructures are strongly correlated. For this reason, in order to optimize the response of the array to maximize the brightness of the thin film, it is key to consider that any change in the morphology of the resonators or the lattice in which they are arranged not only impacts the maximum field enhancement that can be achieved but also the spectral position of the plasmon resonance.

Finally, we invoke the reciprocity theorem to establish a connection between field enhancement numerical simulations and PL enhancement measurements. According to Fermi’s golden rule, the radiative decay rate of a quantum emitter in the electric dipole approximation is proportional to the local density of optical states (LDOS) at its position.^54,55^ LDOS gives information about the number of optical modes available for the emitter to decay. Hence, if an emitter is placed in the regions of high LDOS, it is more likely that it emits. If this quantity is low, the transition rate of the emitter will reduce because the optical environment hinders light generation from that particular spatial position and for that particular frequency. Reciprocity theorem states that it is possible to exchange the source and detector, and therefore, a good receiver also behaves as a good emitter. This implies that the calculations of electric near-field intensity in a photonic system upon illumination allow us to identify spatial regions from where the emission of light will be favored. In view of this, phosphor nanoparticles located in the regions of large field intensity at the emission wavelength should emit brighter than others that sit in the regions where the field is lower. Thus, emitters in close proximity of the antennas should feature enhanced PL compared to those in a reference film, as shown in Figure 3c,d. We calculate the integrated intensity enhancement (IIE) at a given position , which is defined as the field intensity integrated in the volume where nanophosphors are distributed atop the antenna array divided by the same quantity calculated for a reference film of the same thickness without antennas—see Figure 4g.
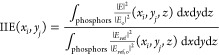


Figure 4h displays a 2D plot in which the IIE is integrated in the vertical coordinate, while we maintain a similar in-plane resolution to that shown by the PL map in Figure 3b. Bright spots of large IIE are found in the spatial positions where the antennas sit. Furthermore, the highly localized character of the resonance along with the large spatial averaging done to compare with the experimental measurements yield significantly lower field intensity values than those shown in Figure 4e,f. In particular, we estimate a maximum IIE of ∼sixfold in the position of the half spheres—see Figure 4h. Thus, our analysis indicates that the emission enhancement observed originates from a combination of resonant excitation and enhanced radiative coupling of few nanophosphors located in close proximity of gold antennas that support an LSPR with a large field enhancement.

## Conclusions

We have developed a simple and nonexpensive method to fabricate large-area metal nanoresonators using a mask made of a monolayer of polymer beads arranged in a triangular lattice. Colloidal lithography allows the fabrication of gold half spheres that support LSPRs, which spectrally overlap with the emission band of GdVO_4_:Eu^3+^ nanocrystals. At resonance, the electric field intensity is largely enhanced in the vicinity of the metal–dielectric interface, which influences greatly the emission of Eu^3+^ cations located nearby. As a result, we demonstrate a 12-fold emission enhancement in ultrathin layers that originates from enhanced radiative coupling of nanophosphors located in close proximity of the gold resonators. Direct patterning of nanophosphor layers with metal nanostructures that behave as optical antennas using colloidal lithography renders ultrathin nanophosphor layers into bright color converters. Our results offer new opportunities for the integration of RE-based coatings with tailored emission properties in compact light-emitting devices of interest for lighting, sensing, or security.

## Supporting Information Available

PLQY measurements; SEM images of gold nanostructure arrays with two different periodicities; SEM images of the gold nanostructures before and after thermal processing; experimental *R*(S4) and 1–*R*–*T* (S5) spectra of the samples after each fabrication step; confocal scanning microscopy measurements; time-dependent PL measurements along with their fittings and residuals; fitting parameters; FDTD simulated *T* spectra; FDTD simulated spatial distribution of the near-field intensity in a unit cell of the array for two wavelengths; FDTD simulated *T* spectra of two arrays of tetrahedra with two different periodicities; FDTD simulated spatial distribution of the near-field intensity in a unit cell of the array of gold hemispheres and tetrahedra; and experimental and calculated *R* and *T* spectra of a 250 nm-thick phosphor nanoparticle layer (PDF)
