# Supplementary material for: Nanoantennas Patterned by Colloidal Lithography for Enhanced Nanophosphor Light Emission
Source: ACS Appl Nano Mater. 2022 Nov 11;5(11):16242–9. doi: 10.1021/acsanm.2c03258 (PMC9706497; doi:10.1021/acsanm.2c03258)
Supplement: Supplementary file 1 — an2c03258_si_001.pdf [file an2c03258_si_001.pdf]

Supporting Information of:

Nanoantennas Patterned by Colloidal Lithography for Enhanced Nanophosphor Light Emission

Jose M. Viaña,[1] Manuel Romero,[1] Gabriel Lozano,[1]* Hernán Míguez[1]*

[1] Instituto de Ciencia de Materiales de Sevilla, Consejo Superior de Investigaciones Científicas-Universidad de Sevilla, C. Américo Vespucio 49, 41092, Sevilla, Spain

Email: g.lozano@csic.es; h.miguez@csic.es

Absolute photoluminescence quantum yield (PLQY) of a material is estimated as the ratio of the number of photons emitted, to the number of photons absorbed. Our films were excited at 285 nm and the emission and scattering peaks measured in the integrating sphere in the spectral range comprised between 270 nm and 850 nm (see red curve in Figure S1). In addition, a scattering sample was also measured as a reference (see black curve in Figure S1). Notice that the PL spectrum is scaled in the emission spectral range to correct for the background (see gray curve in Figure S1). Hence, the PLQY of the film is calculated as

$$PLQY = (E-E_b)/(S_{ref}-S)$$

where E, E_b, S_{ref} and S are the integrated intensities of emission and scattering peaks for the sample and the reference, as illustrated in Figure S1. As a result, we obtain PLQY values that go from 0.07 for as-deposited films to 0.55 after thermal processing at 450 ºC.

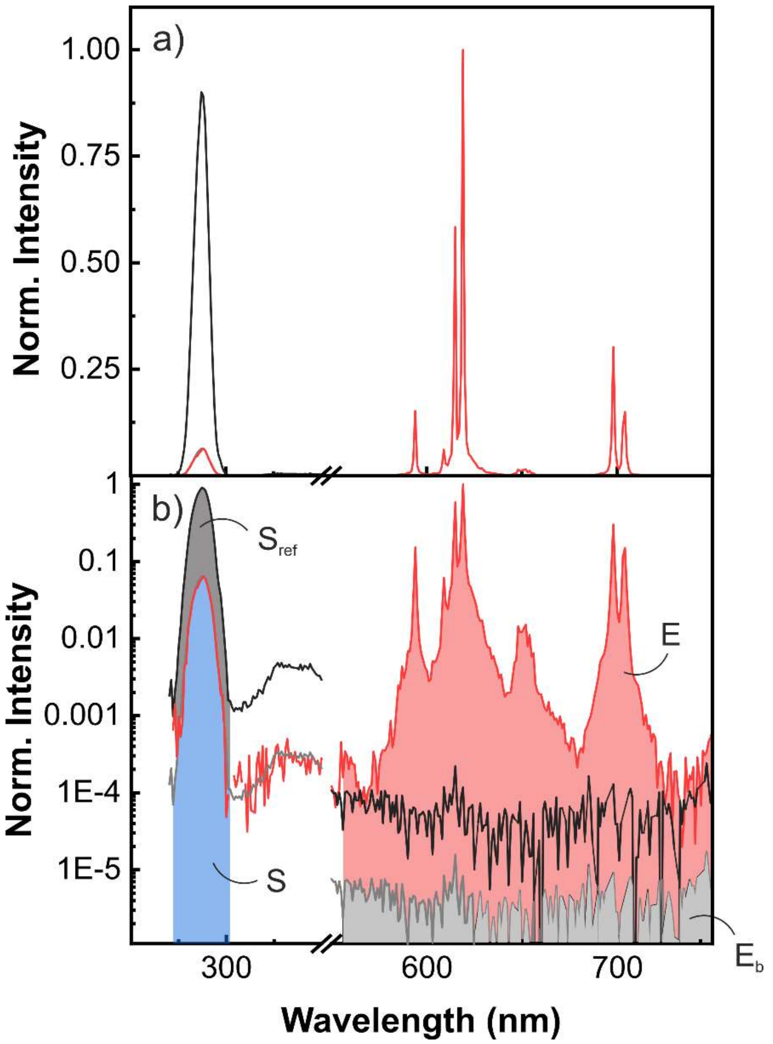

__Figure S1__. (a) Intensity spectrum of a nanophosphor film annealed at 450 ºC (red) and that of a scattering sample that serve as reference (black). (b) Intensity spectra shown in (a) displayed in log scale along with the background-corrected spectrum of the film in the emission range (gray).

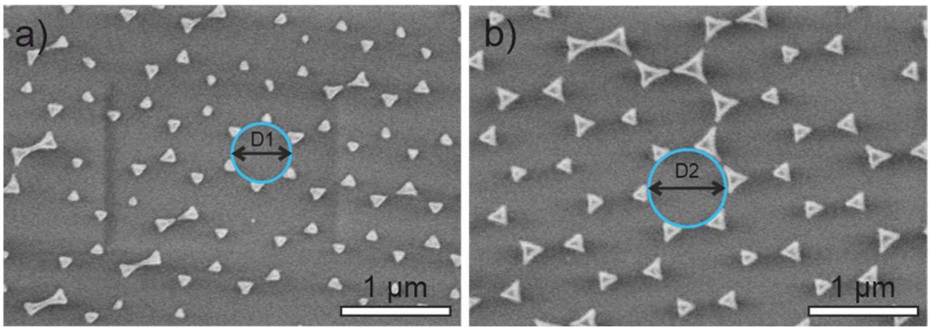

Figure S2. *SEM images of the top view of gold nanostructure arrays fabricated from colloidal masks of sphere size (a)* $560\ nm$ *and* (b) $720\ nm$.

As-deposited nanoparticles show tetrahedral shape with rounded edges and corners (see Figure S3a-b), which shift to halfsphere-like shape with thermal treatment (see Figure S3c-d). Similar behavior is observed when a thin nanophosphor film is deposited atop the gold nanostructures. Nanostructures that initially feature a tetrahedral shape become more rounded, like hemispheres, after thermal processing, as shown in Figure S3 (e-f).

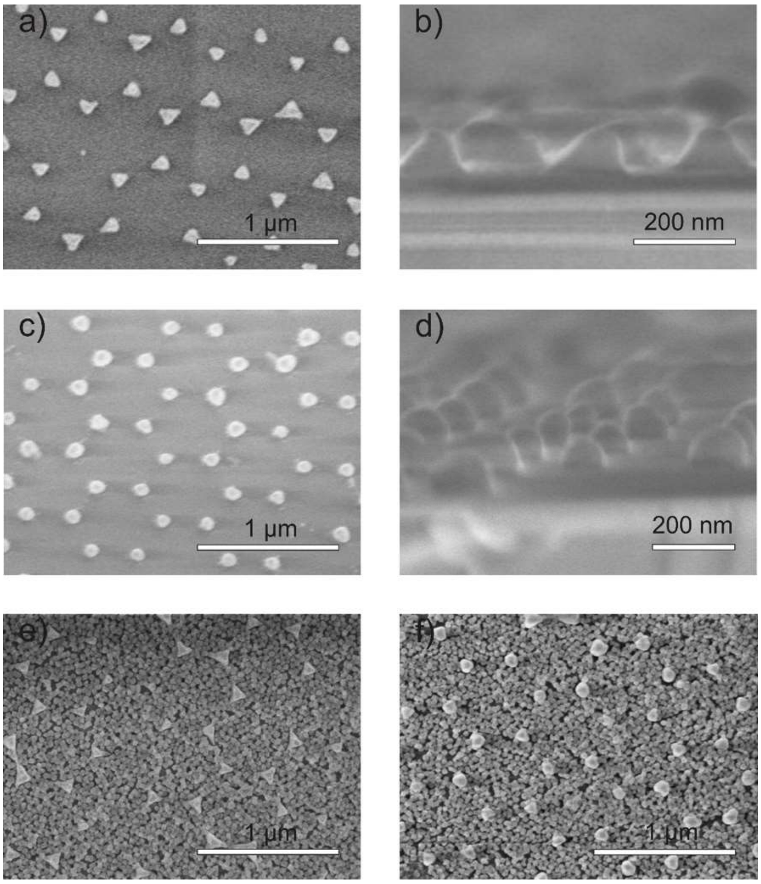

Figure S3. *(a-d) Scanning electron microscopy (SEM) pictures of a top view (a,c) and a cross section (b,d) of an array of gold antennas deposited over a glass substrate before (a-b) and after thermal processing (c-d). (e-f) SEM pictures of the top view of a nanophosphor film deposited atop the gold nanoparticle array before (e) and after thermal processing (f).*

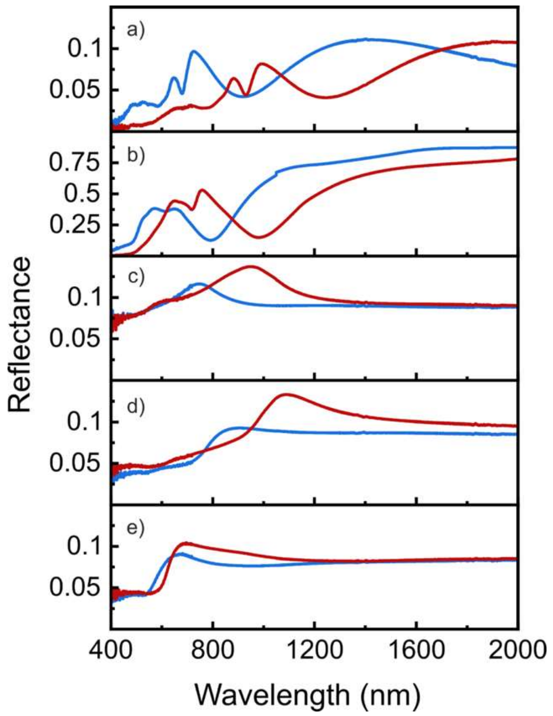

Figure S4. *Experimental specular reflectance spectra of the (a) colloidal mask, (b) colloidal mask over which 100 nm of gold was deposited, (c) periodic array of gold nanostructures, (d) same array after* $GdVO_4:Eu^{3+}$ *nanophosphor thin film deposition, and (e) after thermal processing. Blue (red) line correspond to samples prepared using a colloidal mask made of polymer spheres with D = 560 nm (720 nm).*

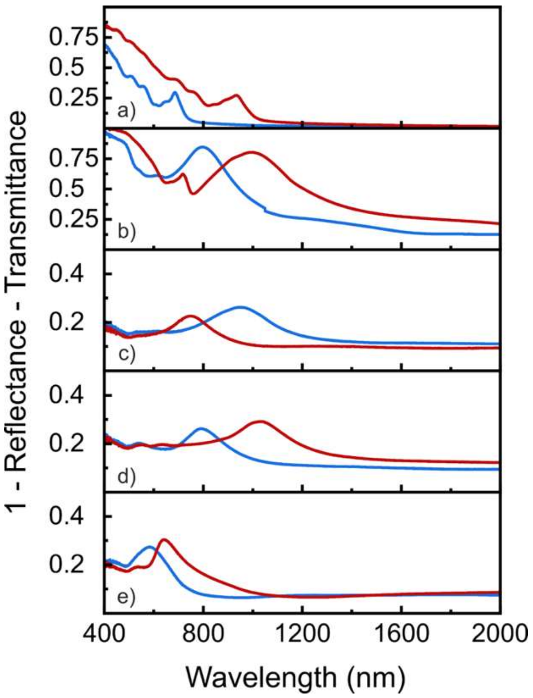

Figure S5. *Experimental 1-R-T spectra of the (a) colloidal mask, (b) colloidal mask over which 100 nm of gold was deposited, (c) periodic array of gold nanostructures, (d) same array after* $GdVO_4:Eu^{3+}$ *nanophosphor thin film deposition, and (e) after thermal processing. Blue (red) line correspond to samples prepared using a colloidal mask made of polymer spheres with D = 560 nm (720 nm).*

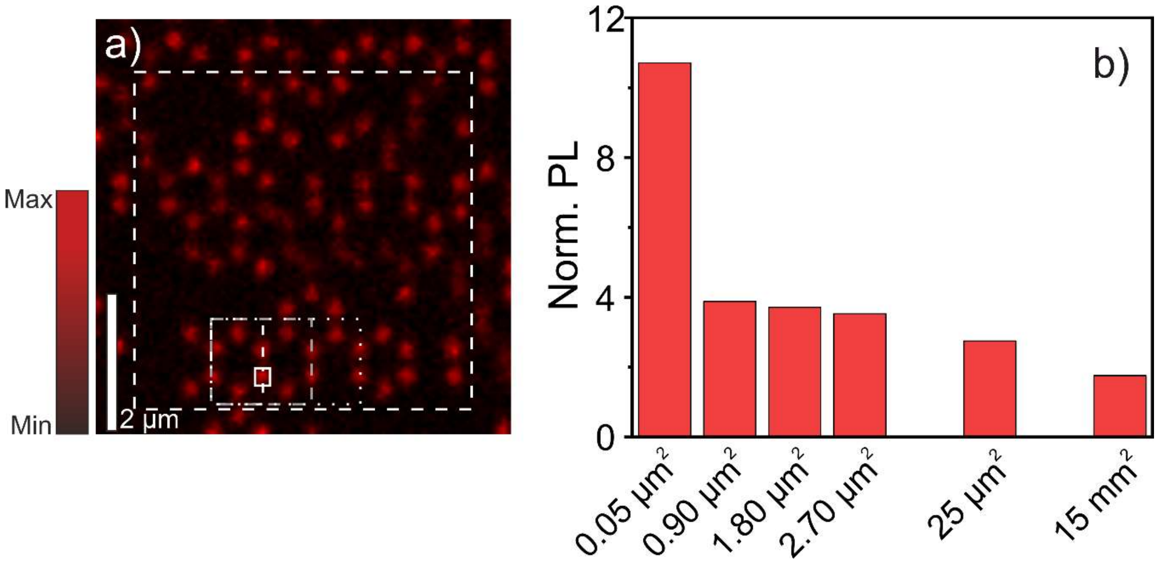

Figure S6. *(a) Confocal scanning microscopy image of the Photoluminescence (PL) of a $GdVO_4:Eu^{3+}$ nanophosphor thin film deposited over an array of gold nanostructures. Light is integrated in the spectral range comprised between $\lambda = 606$ nm and $\lambda = 636$ nm. PL intensity integrated over the different areas indicated in (a): 0.05 μm^2, 0.90 μm^2, 1.80 μm^2 , 2.70 μm^2, 25 μm^2. For comparison, the emission from a 15 mm^2 area measured in a spectrofluorometer is also displayed in the same graph. PL is normalized to the same quantity measured from the corresponding area of a reference film devoid any metal.*

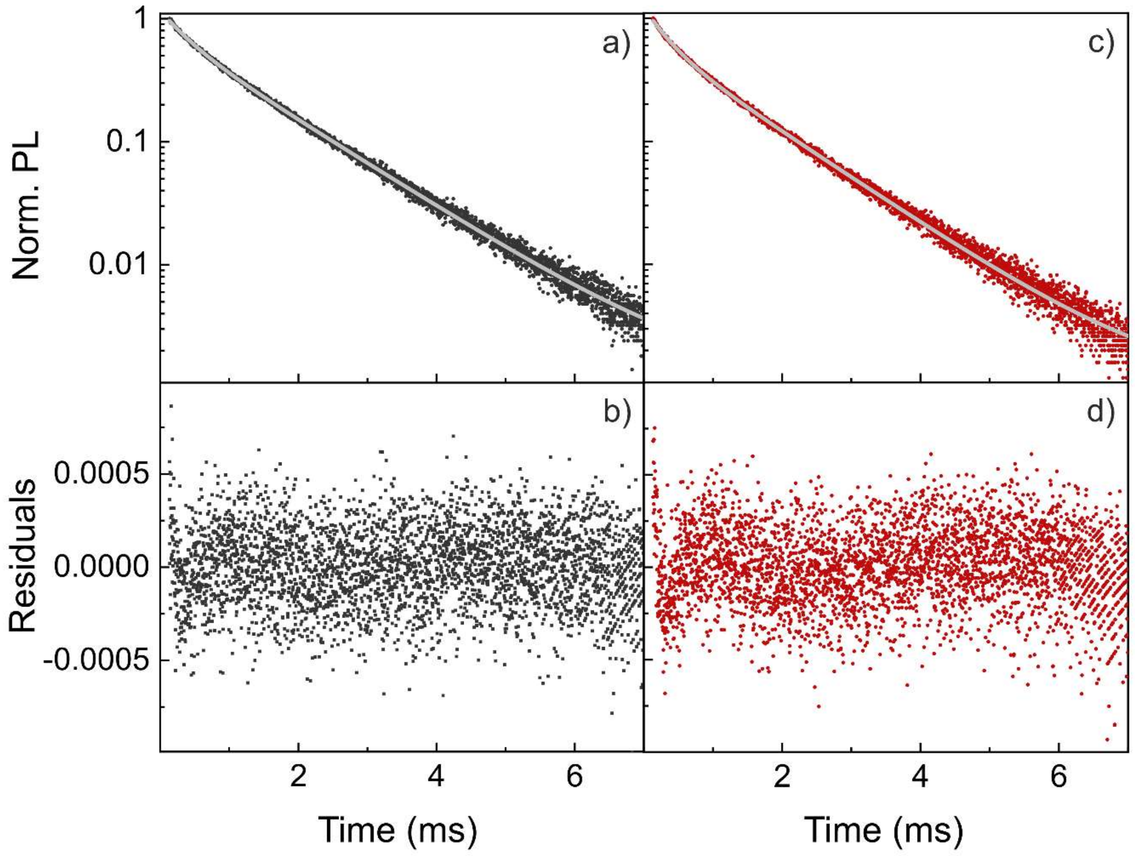

Figure S7. *Time-dependent PL monitored at $\lambda = 620$ nm from a thin nanophosphor film deposited over the gold array (red dots) (a), and a flat substrate (black dots) (c). Fittings are also shown as light grey curves. Corresponding residuals are displayed in (b) and (d), respectively.*

sample	A_1	A_2	τ_1 (ms)	τ_2 (ms)	τ_{aver} (ms)
Reference	1412 (±20)	3396 (±22)	0.36 (11%)	1.2 (89%)	1.16
Antennas	1785 (±16)	2986 (±17)	0.31 (14%)	1.2 (86%)	1.02

Table S1. *Fitting parameters.*

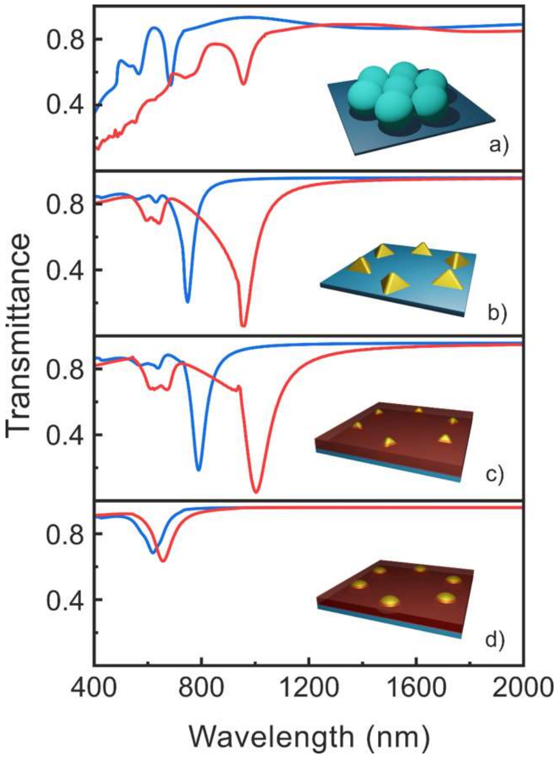

Figure S8. *FDTD simulated ballistic transmittance spectra of a (a) monolayer of spheres of D = 720 (red) and D = 560 nm (blue), (b) periodic array of gold tetrahedra, (c) same arrays with a layer of 35 nm of dielectric with n=1.3, and (d) periodic array gold halfspheres covered by a 35 nm dielectric layer with n=1.3. Blue (red) line correspond to samples prepared using a colloidal mask made of polymer spheres with D = 560 nm (720 nm). 3D sketches of the different structures are shown as insets.*

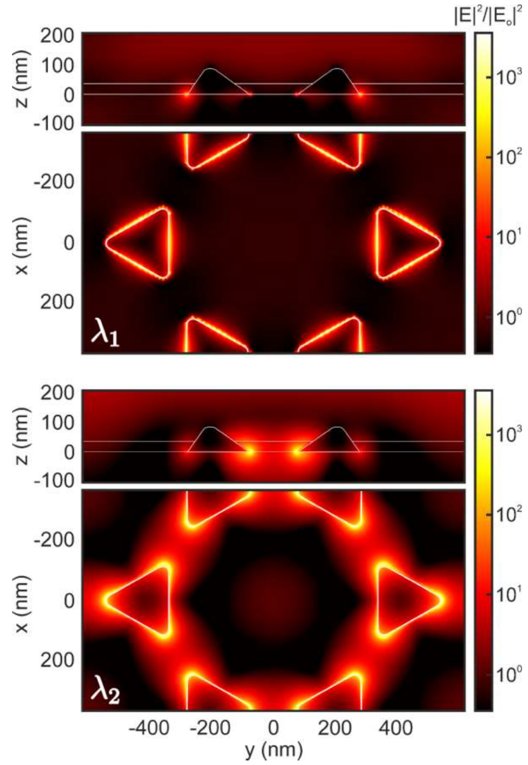

Figure S9. *FDTD simulated spatial distribution of the near-field intensity in an array of gold tetrahedra on a substrate covered by a dielectric film in a unit cell of the array for 2 different wavelengths:* $\lambda_1 = 620\,nm$ *and* $\lambda_2 = 1016\,nm$*. XY plane is shown for Z=0nm, while YZ plane intersects through the center of two nanoantennas.*

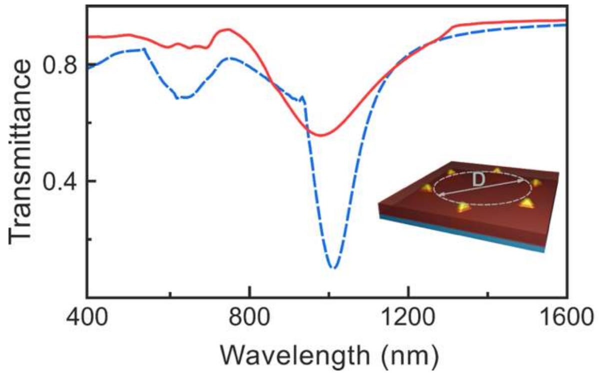

Figure S10. *FDTD simulated transmitted spectra of two arrays of tetrahedra with periodicity* $D = 720nm$ *(blue) and* $D = 1000nm$ *(red). The size and shape of the individual antennas are kept unchanged*

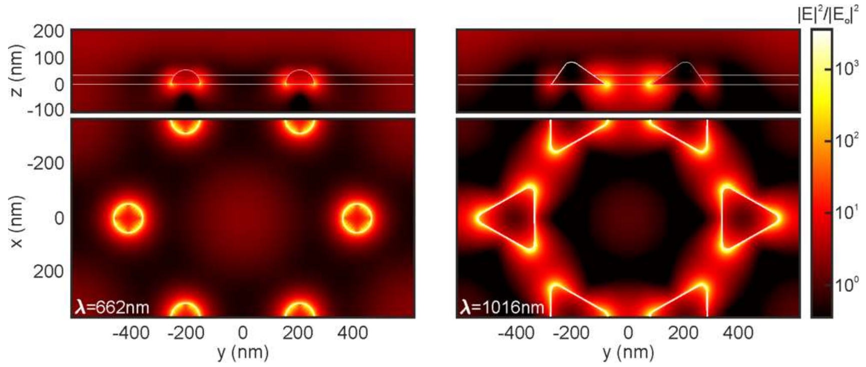

***Figure S11**. Comparison of the FDTD simulated spatial distribution of the near-field intensity in an array of gold hemispheres (left) and tetrahedra (right) on a substrate covered by a dielectric film in a unit cell of the array at the resonant wavelength of each system.*

.

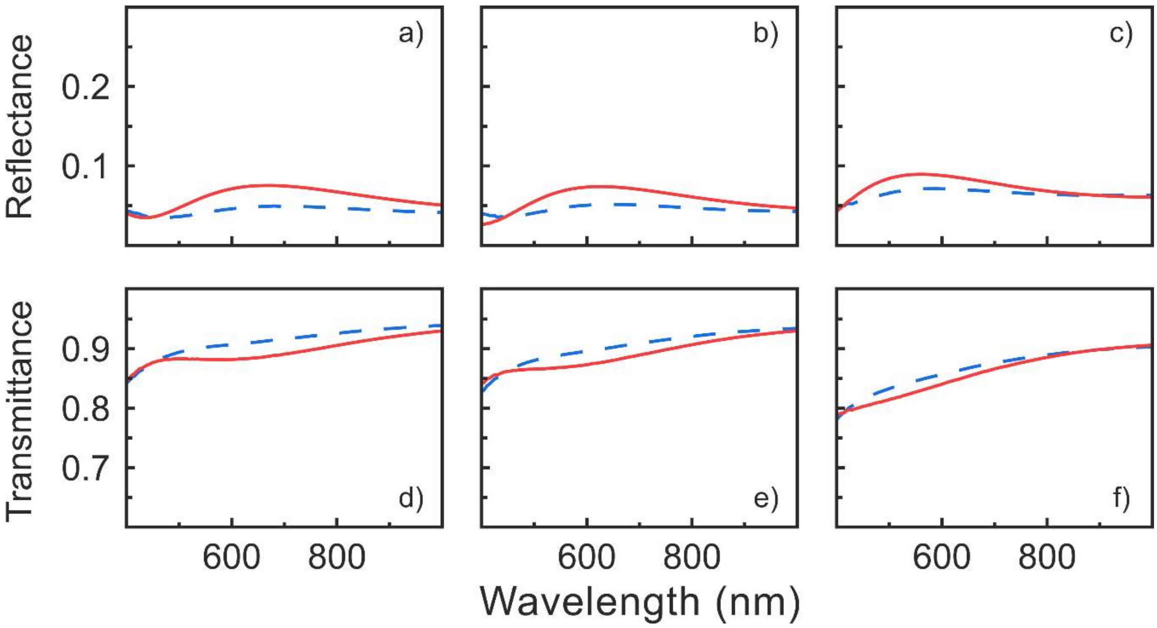

Figure S12. *Experimental (dashed curves) and calculated (solid curves) (a-c) specular reflectance and (d-f) ballistic transmittance of a 250 nm-thick nanophosphor layer for three different incident angles: 6 deg (a, d), 30 deg (b, e) and 50 deg (c, f). Calculations consider a refractive index of 1.3 for the nanophosphor layer.*
